# Pancreas MRI Segmentation Into Head, Body, and Tail Enables Regional Quantitative Analysis of Heterogeneous Disease

**DOI:** 10.1002/jmri.28098

**Published:** 2022-02-07

**Authors:** Alexandre Triay Bagur, Paul Aljabar, Gerard R. Ridgway, Michael Brady, Daniel P. Bulte

**Affiliations:** ^1^ Department of Engineering Science University of Oxford Oxford UK; ^2^ Perspectum Ltd Oxford UK

**Keywords:** segmentation, groupwise registration, NAFPD, diabetes, MRI‐PDFF, heterogeneity

## Abstract

**Background:**

Quantitative imaging studies of the pancreas have often targeted the three main anatomical segments, head, body, and tail, using manual region of interest strategies to assess geographic heterogeneity. Existing automated analyses have implemented whole‐organ segmentation, providing overall quantification but failing to address spatial heterogeneity.

**Purpose:**

To develop and validate an automated method for pancreas segmentation into head, body, and tail subregions in abdominal MRI.

**Study Type:**

Retrospective.

**Subjects:**

One hundred and fifty nominally healthy subjects from UK Biobank (100 subjects for method development and 50 subjects for validation). A separate 390 UK Biobank triples of subjects including type 2 diabetes mellitus (T2DM) subjects and matched nondiabetics.

**Field strength/Sequence:**

A 1.5 T, three‐dimensional two‐point Dixon sequence (for segmentation and volume assessment) and a two‐dimensional axial multiecho gradient‐recalled echo sequence.

**Assessment:**

Pancreas segments were annotated by four raters on the validation cohort. Intrarater agreement and interrater agreement were reported using Dice overlap (Dice similarity coefficient [DSC]). A segmentation method based on template registration was developed and evaluated against annotations. Results on regional pancreatic fat assessment are also presented, by intersecting the three‐dimensional parts segmentation with one available proton density fat fraction (PDFF) image.

**Statistical Test:**

Wilcoxon signed rank test and Mann–Whitney *U*‐test for comparisons. DSC and volume differences for evaluation. A *P* value < 0.05 was considered statistically significant.

**Results:**

Good intrarater (DSC mean, head: 0.982, body: 0.940, tail: 0.961) agreement and interrater (DSC mean, head: 0.968, body: 0.905, tail: 0.943) agreement were observed. No differences (DSC, head: *P* = 0.4358, body: *P* = 0.0992, tail: *P* = 0.1080) were observed between the manual annotations and our method's segmentations (DSC mean, head: 0.965, body: 0.893, tail: 0.934). Pancreatic body PDFF was different between T2DM and nondiabetics matched by body mass index.

**Data Conclusion:**

The developed segmentation's performance was no different from manual annotations. Application on type 2 diabetes subjects showed potential for assessing pancreatic disease heterogeneity.

**Level of Evidence:**

4

**Technical Efficacy Stage:**

3

Pancreas pathology, such as fatty infiltration, diabetes, chronic pancreatitis, and pancreatic cancer, is rising rapidly with the increasing prevalence of obesity and the metabolic syndrome.^1^ Obesity leads to ectopic fat deposition in organs like the heart, liver, and pancreas.^1^ While nonalcoholic fatty liver disease (NAFLD) is a well‐recognized disease entity, now affecting one fourth of the worldwide population and one third of US adults,^2^ nonalcoholic fatty pancreas disease (NAFPD) was only coined relatively recently^1^, ^3^ despite showing similar prevalence described in a recent meta‐analysis.^4^ Analogously to NAFLD, NAFPD triggers inflammatory processes that, if left untreated, may lead to chronic pancreatitis and pancreatic cancer.^5^, ^6^ NAFPD has also been linked to type 2 diabetes.^7^, ^8^ Early detection of pancreatic disease is therefore important; however, these are often “silent” conditions that only become symptomatic at a late stage, when they may already be irreversible.^5^ Incidental findings, where the target organ is near the pancreas, for instance, in quantitative imaging of the liver, potentially offer a way to detect pancreas pathology early.

Pancreatic disease processes, including fat infiltration, fibro‐inflammation, and pancreatic cancer, are also spatially inhomogeneous.^9^, ^10^ There is increasing interest in studying pancreatic disease and the implications of disease heterogeneity, aiming to describe regional differences and localize pancreatic lesions. Early work using computed tomography (CT) classified uneven pancreatic fat infiltration into multiple subtypes or patterns, depending on the affected regions.^10^ Uneven distribution of islet cells that are responsible for insulin secretion and blood sugar regulation has been reported using histology.^11^ Fibrosis has been more commonly found in the ventral pancreas than in the dorsal pancreas in patients with ampullary carcinoma.^12^ The frequency of pancreatic cancer also differs regionally, with 60%–70% occurrence in the head of the pancreas, and the symptoms also vary by location.^13^, ^14^ From the imaging modalities commonly used for pancreatic assessment, including histology, endoscopic ultrasound, contrast‐enhanced CT, and MRI, only MRI can provide safe, noninvasive quantitative information of pancreas state, while providing full coverage and measures of spatial heterogeneity. Quantitative MRI biomarkers such as proton density fat fraction (PDFF) and T_1_ have shown potential in detecting pancreas steatosis and early‐stage chronic pancreatitis, respectively^15^, ^16^; PDFF has been used for longitudinally monitoring total pancreatic fat deposition in a diabetes remission trial.^17^ Apparent diffusion coefficient from diffusion‐weighted imaging has shown potential at grading malignancy of a certain pancreatic neoplasm type.^18^ While some studies using MRI have reported clinically important quantitative differences between pancreas subsegments,^19^, ^20^ other studies have not found such differences.^21^

The pancreas is anatomically divided into three segments: head, body, and tail. The pancreas head sits within a C‐shaped structure formed by the duodenum and joins with the pancreas body via the pancreas neck, a narrowing or “isthmus” that bends around the superior mesenteric vessels. The pancreas neck is typically approximately 2 cm long and is commonly included as part of the head. The pancreas body spans from the left border of the superior mesenteric vein to the left border of the aorta, where it is joined to the tail. It is generally considered that the body–tail boundary is at the midpoint lengthwise of the two segments.^22^ Other pancreas subsegment classification systems have been proposed for the purposes of surgical resection based on embryological foundations.^22^, ^23^ Most studies of pancreas pathology using MRI have analyzed the images using regions of interest (ROIs), particularly a standard 3‐ROI placement strategy targeting pancreatic head, body, and tail,^20^, ^21^, ^24^, ^25^ although some have placed an extra ROI in the pancreatic neck.^19^ While ROIs have the advantage of avoiding artifactual regions, their choice of placement inevitably adds interobserver variation that may obscure clinically important differences between pancreatic segments.

Pancreas segmentation that aims to delineate the whole organ through the use of two‐dimensional or volumetric scans has been proposed as an alternative analysis method to the 3‐ROI placement strategy, which may improve observer‐dependent bias and provide more advanced metrics for the spatial assessment of chronic disease.^26^ Pancreas segmentation can be performed using widely differing amounts of user intervention, however, such is the variability in size and shape of the pancreas that it is often considered too tedious to manually delineate in practice. Manual segmentation is also too costly and generally infeasible in large databases such as the UK Biobank.^27^ Metrics derived from pancreas segmentations are clinically important, for instance, total pancreatic volume or the irregularity of the pancreas contour in the context of diabetes.^28^, ^29^ Pancreas segmentations may also be used for subsequent characterization of the pancreas in functional or structural quantitative imaging data acquired separately during the same imaging session.

Automated pancreas segmentation methods that have been proposed to date have been based on traditional multiatlas methodology or, more recently, convolutional neural networks.^30^, ^31^ However, while these may provide whole‐organ measurements, they do not characterize disease regionally by pancreas subsegments. One automated method for pancreas subsegmentation was reported based on *k*‐means clustering^32^ that was applied to pancreas motion analysis under radiation therapy. However, this method is dependent on initial seed points and multiple images from multiple breathing phases and was not validated for accuracy. For these reasons, the validation of a robust, automated approach for pancreas subsegmentation is desirable, with potential to bridge the gap between currently available technology and standard clinical assessment.

Starting from a segmentation of a whole‐organ, landmark‐based approaches have been proposed for subsegmentation into the organ's constituent parts, eg, the Couinaud segments in the case of the liver, where “landmarks” define planes of separation between the liver segments.^33^ However, landmark localization is relatively sensitive to noise and overall image quality. Other methods have addressed organ subsegmentation as a single task, in which segmentation models create a multilabel segmentation, each label corresponding to an individual subsegment. For example, atlas‐based segmentation uses image registration to propagate labels from a probabilistic template (constructed offline) to a target dataset.^34^ Multiatlas segmentation (MAS) or deep learning (DL) segmentation methods may also be used; however, these typically need individual annotations on training subjects and may require large amounts of annotations.^34^ Some DL methods have drawn inspiration from traditional atlas‐based methodology.^35^

Thus, the aims of this study were to:

1. develop and validate an automated imaging‐based method for pancreas subsegmentation and
2. show initial application of the method in regional assessment of pancreatic disease.

## Materials and Methods

First, the data that were used for template creation are described together with preprocessing of the training and validation data. Then, a groupwise registration‐based parts segmentation method is presented, and the validation experiment is described. Finally, the application of the method to a type 2 diabetes cohort of UK Biobank is shown.

### MRI Data

MRI data from the UK Biobank imaging substudy were used.^27^ UK Biobank received ethical approval from the North West Multi‐center Research Ethics Committee, and written informed consent was obtained for all subjects. One hundred subjects were used for method development, 44 females and 56 males. All were nominally healthy subjects aged 50–70 with a mean age of 55 years for females and 57 years for males. The mean body mass index (BMI) was 25.5 kg/m^2^ for females and 27.1 kg/m^2^ for males. An additional 50 UK Biobank subjects were used for validation, 21 males and 29 females, with a mean age of 53 years and 57 years and a BMI of 25.9 kg/m^2^ and 26.5 kg/m^2^, respectively.

As an initial exploration of fat heterogeneity in diseased subjects, a separate dataset of UK Biobank subjects was developed, comprising 390 triples of 1) self‐reported type 2 diabetes mellitus (T2DM) subjects; 2) gender‐, age‐, and BMI‐matched nondiabetic subjects; and 3) gender‐ and age‐matched nondiabetic subjects with chosen BMI of <25 kg/m^2^. These groups of subjects will be referred to as: *T2DM*, *matched high BMI nondiabetics*, and *matched low BMI nondiabetics* throughout this work. A total of 390 × 3 = 1170 subjects were selected. Age was matched to within 5 years, and BMI was matched within one point in all cases. The mean age and the mean BMI for the three groups were 57 years and 31.0 kg/m^2^, respectively, for T2DM; 57 years and 30.8 kg/m^2^, respectively, for Matched high BMI nondiabetics, and 56 years and 23.0 kg/m^2^, respectively, for matched low BMI nondiabetics.

All subjects had been scanned with a 1.5 T Siemens Aera scanner (Siemens Healthineers, Erlangen, Germany) using a two‐point Dixon protocol covering neck to knee, acquired using six overlapping slabs and uploaded to the UK Biobank as Data‐Field 20201. Each slab was acquired using TE = 2.39/4.77, TR = 6.69 msec, 10° flip angle, and pixel bandwidth = 440 Hz. Only datasets from the first imaging session of UK Biobank (instance 2) were used. Slabs were stitched together, and the resulting neck‐to‐knee volume was cropped to the abdominal region, resulting in a subvolume that generally included slabs 2–4 (more details are available in the study by Owler et al^36^). Slabs 2–4 each had a reconstructed voxel size of 2.23 mm × 2.23 mm × 4.5 mm, an image matrix size of 224 × 174 with 44 slices, a phase resolution percentage of 71%, and a slice resolution percentage of 100%.

Multiecho gradient‐recalled echo (GRE) two‐dimensional single‐slice data were also obtained from a separate breath‐hold scan for the calculation of confounder‐corrected PDFF maps, uploaded to the UK Biobank as Data‐Field 20260. The GRE scan had a reconstructed voxel size of 2.5 mm × 2.5 mm × 6 mm and an image matrix size of 160 × 160, 10 echoes, TE_1_ = ΔTE = 2.38 msec, TR = 27 msec, 20° flip angle, and pixel bandwidth = 710 Hz. A confounder‐corrected magnitude‐based chemical‐shift encoding method^37^ was used to reconstruct PDFF maps from the raw 10‐echo GRE data, which uses a multi‐peak spectral model from liver fat^38^ and accounts for $R_{2}^{*}$ decay.

**FIGURE 1:**
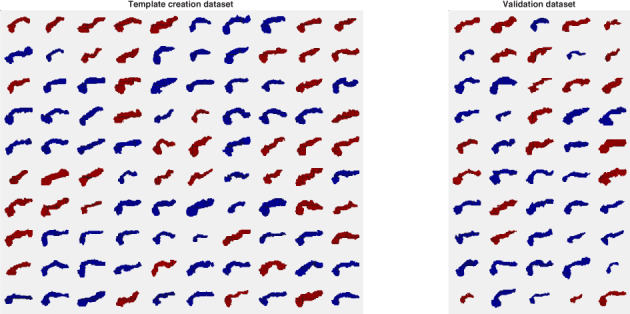
Manual whole‐pancreas segmentations from the template construction (“training”) dataset and the validation dataset, sorted by subjects' age from youngest to oldest (females in red and males in blue).

The whole pancreas had been delineated manually on all 150 training and validation datasets by AB (5 years of experience) as part of previous work.^36^ Figure 1 shows three‐dimensional renderings of the whole‐pancreas segmentations for all subjects in both the template creation dataset and the validation dataset. The volumes and the corresponding whole‐organ segmentations were resampled to 2 mm isotropic resolution. We also minimally co‐registered the subjects by translating them to align their centroids. The centroid of subject 1 was arbitrarily used as a reference. The prealignment provided a better starting point for the nonlinear registration algorithm, both for template creation and for method inference. Currently, the software is compatible with images that are isotropic, have identical size and are in approximate alignment with each other.

### Method Description

**FIGURE 2:**
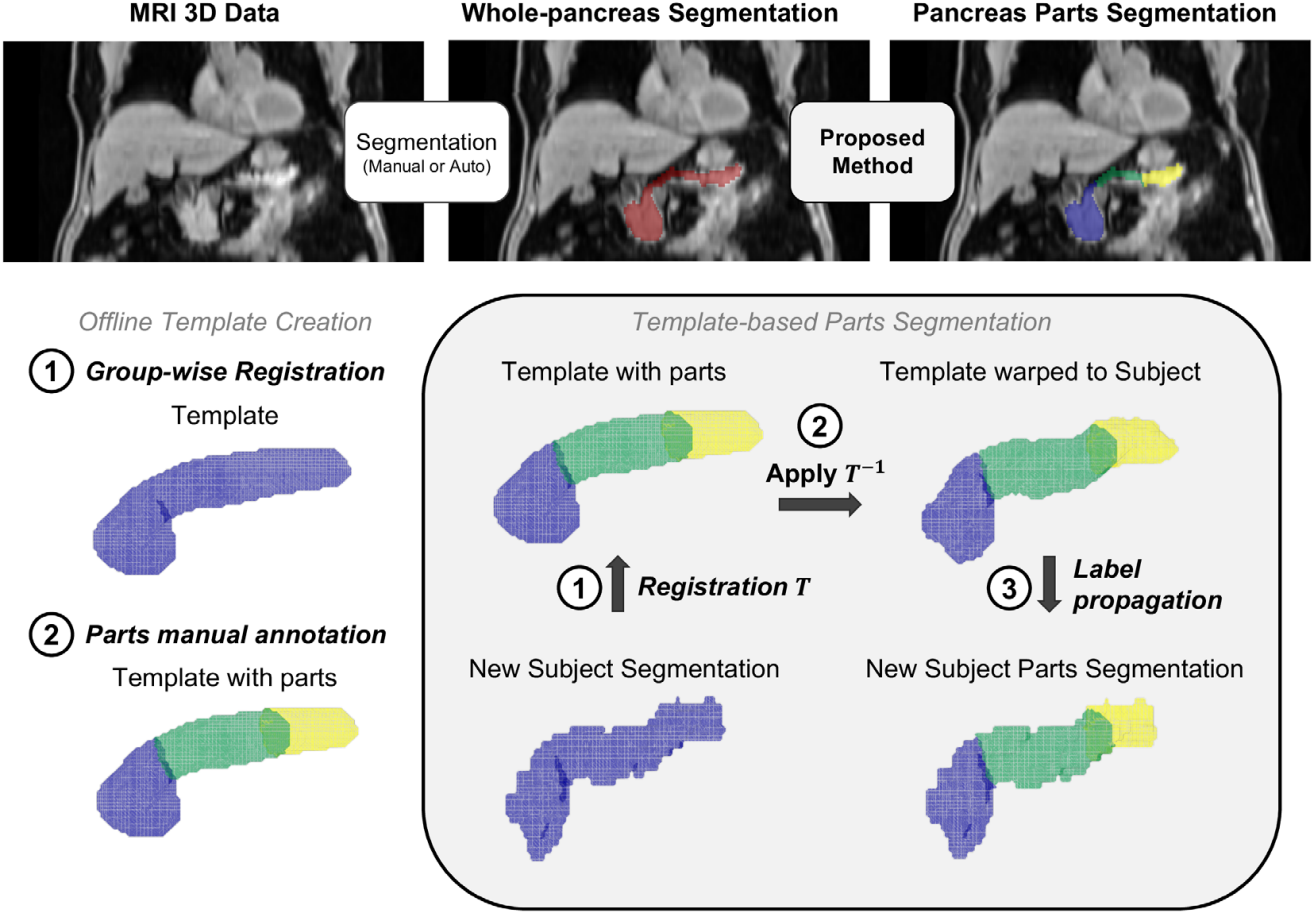
Method description. (top) Overall pipeline for whole‐pancreas segmentation and parts segmentation. (bottom‐left): (1) Offline groupwise registration of the whole‐pancreas segmentation generated a population average (“template”), on which (2) the parts were manually annotated (“parts template,” head: blue, body: green, tail: yellow). (bottom‐right) For a new subject, the method (1) computes a registration transformation from the subject's segmentation to the template, (2) applies the inverse transformation on the parts template, and (3) propagates the warped parts template labels to the segmentation.

An overview of the groupwise registration method is shown in Fig. 2. The method takes a whole‐pancreas segmentation as input, either delineated manually or with an automated approach; in this part of the study, the input whole‐pancreas segmentations were manually obtained. First, an average pancreas template is constructed offline using groupwise registration from the *N* = 100 method development dataset of manual whole‐pancreas segmentations. Then, the pancreas parts (head, body, and tail) are manually annotated on the constructed template, resulting in a pancreas parts template. Method inference (parts segmentation) is performed by registration of the pancreas parts template to a new target whole‐pancreas segmentation. Then, the registered parts template labels are propagated to the target whole‐pancreas segmentation, obtaining a pancreas parts segmentation for that subject. Offline parts template construction and parts segmentation inference steps are detailed in the following paragraphs.

The backbone for template construction is the large deformation diffeomorphic metric mapping (LDDMM) via geodesic shooting algorithm developed by Ashburner and Friston^39^ and available under the “Shoot” toolbox of the SPM12 software (https://www.fil.ion.ucl.ac.uk/spm/software/spm12/). The toolbox uses diffeomorphic transformations to co‐register all the template construction segmentations iteratively into a population average, i.e. the “template” image. MATLAB R2021a (The MathWorks, Inc, Natick, MA, USA) and the batch processing capability of SPM12 were used to run template creation. A probabilistic template (0–1) was obtained from this step after four iterations, that were binarized by thresholding at 0.5.

Pancreas head, body, and tail were annotated on the template image by AB. Note this template‐based approach enables annotation of parts on the constructed template, instead of annotating each of the training subjects individually, thus requiring a single annotation step. The initial assumption was that this approach would be substantially equivalent to annotating each “training” subject individually. One additional advantage of this annotation strategy was that some salient features appear on the template after groupwise registration, which correspond to the landmarks defining the pancreas subsegments. These landmarks may otherwise be difficult to identify in individual cases, and correct landmark identification is highly dependent on image quality. Annotation was performed by defining one boundary plane between head and body and another boundary plane between body and tail.

Given a whole‐pancreas segmentation for a new subject, which can be either manually delineated or computed automatically, the method first computes a registration transformation from the subject's whole‐pancreas segmentation to the template (again initialized by aligning the centroid). The method then applies the inverse of that registration transformation onto the parts template. Finally, it propagates the labels of the warped parts template toward the whole‐pancreas segmentation, obtaining a parts segmentation for that new subject.

### Validation

The initial manual pancreas segmentations from the validation dataset were subsegmented using the described groupwise registration method and also the method based on *k*‐means proposed by Fontana et al.^32^ The latter was implemented for a single image (single “breathing phase”), choosing the initial cluster centroids using the *k*‐means++ algorithm. A dedicated annotation protocol based on the three‐dimensional “scalpel” tool of ITK‐SNAP (http://www.itksnap.org/)^40^ was developed that demonstrated the manual annotation of a whole‐pancreas segmentation into parts. The protocol gave instructions for the drawing of two separation planes, one plane at the head–body boundary and one plane at the body–tail boundary, both as perpendicular to the pancreas centerline as possible. The protocol was distributed to four separate medical imaging scientists with ranging degrees of experience in annotating abdominal medical images for research: AB (5 years of experience), MB (25 years of experience), PA (10 years of experience), and JR (<1 year experience), namely R1 to R4, to produce reference annotations. R4, who we refer to as *naïve observer*, was a recently hired technologist with no prior experience with pancreas anatomy or pancreas imaging and was included in the study so that the robustness of the annotation protocol to rater experience could be estimated. Of the 50 subjects, 10 were included at random twice in the dataset for the purpose of assessing intraobserver variability (referred to as annotation *a* and annotation *b*). This yielded a total of 60 annotations per rater. Interobserver variability was also assessed by comparing annotations over multiple raters. The interobserver performance may be used as a comparative benchmark for the automatic results.

**FIGURE 3:**
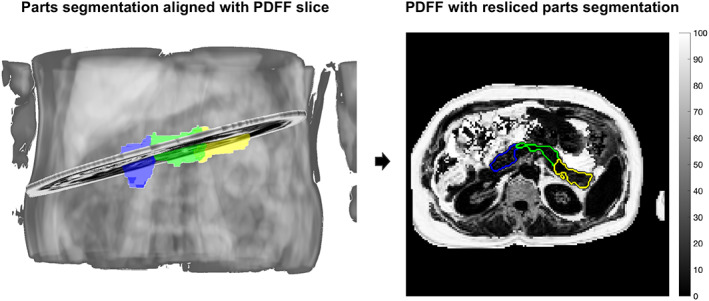
Pancreas segmentation (head: blue, body: green, and tail: yellow) enables quantification of pancreas imaging biomarkers by parts, eg, PDFF, by intersection of the segmentation with the quantitative scan. PDFF = proton density fat fraction

For automated and manual segmentations, the volumes of individual parts were determined, as was the pancreatic fat by parts from PDFF maps (when available). For the latter, the median PDFF values from head, body, and tail were reported after reslicing the parts segmentation onto the reconstructed PDFF map. The three‐dimensional parts segmentation volume was intersected with the two‐dimensional PDFF map using the DICOM Reference Coordinate System information, as illustrated in Fig. 3. Differences in volumes and PDFF between the automated parts segmentation and the manual parts segmentations were reported for each subject. As a quality control (QC) step, segment masks with an area of ≤30 pixels were excluded from the comparisons. The median PDFF of the segment masks was reported after excluding pixels with values exceeding 50%, followed by morphological opening with a disk structuring element of 3 pixels in diameter. The 50% PDFF threshold aimed to exclude nonparenchymal pancreatic tissue, eg, surrounding visceral adipose tissue that could have been introduced due to slight subject motion between breath‐holds. Individual segments were excluded from further statistical analysis if they did not meet these QC criteria.

### Pancreatic Fat Quantification by Parts in Type 2 Diabetes

Since manual whole‐pancreas delineations were not available for these subjects, automated whole‐pancreas segmentations obtained previously in the study by Owler et al^36^ were used, computed using the Attention U‐Net model based on the work of Schlemper et al.^31^ The groupwise registration‐based automated parts segmentation method was run on the automated whole‐pancreas segmentations. The reslicing plus QC approach explained in the previous section was run to measure median fat accumulation in the pancreatic head, body, and tail. Pancreatic fat quantification by parts was compared between the three subject groups.

### Statistical Analysis

Direct validation of automated pancreas subsegmentation was performed using generally accepted segmentation performance metrics, namely Dice similarity coefficient (DSC) and 95th percentile Hausdorff distance (95%HD), as well as the reported volume of each part. Intraobserver agreement, interobserver agreement, and “manual vs. automated” agreement were evaluated using Bland–Altman analysis and right‐tailed Wilcoxon signed rank statistical testing. For the 10 subjects used in intrarater variation assessment, the following comparisons were generated for the experienced raters and combined: R1a vs. R1b, R2a vs. R2b, R3a vs. R3b, yielding a total of 30 datapoints (10 subjects × 3 raters). Intrarater agreement of the inexperienced rater R4, R4a vs. R4b was reported separately and compared to intrarater agreement of R1–R3. For interobserver variation assessment, three comparisons among raters were combined, R1 vs. R2, R1 vs. R3, R2 vs. R3, with 50 parts segmentations in each comparison, yielding 150 datapoints. The robustness of the annotation protocol was tested for rater experience by comparing the segmentation performance in terms of DSC overlap of the naïve observer R4 vs. themselves and vs. R1–R3. For manual vs. automated (*Auto*), the following comparisons were performed and combined for each automated method separately: R1 vs. Auto, R2 vs. Auto, R3 vs. Auto, each with 50 parts segmentations, that resulted in 150 datapoints.

Indirect validation was performed by evaluating agreement between automated and manual parts segmentation quantification of PDFF using Bland–Altman analysis and the Wilcoxon signed rank test. Mann–Whitney *U*‐test was used to compare quantification by parts across groups of subjects with and without type 2 diabetes.

A *P* value < 0.05 was considered to be statistically significant.

## Results

### Direct Validation

**FIGURE 4:**
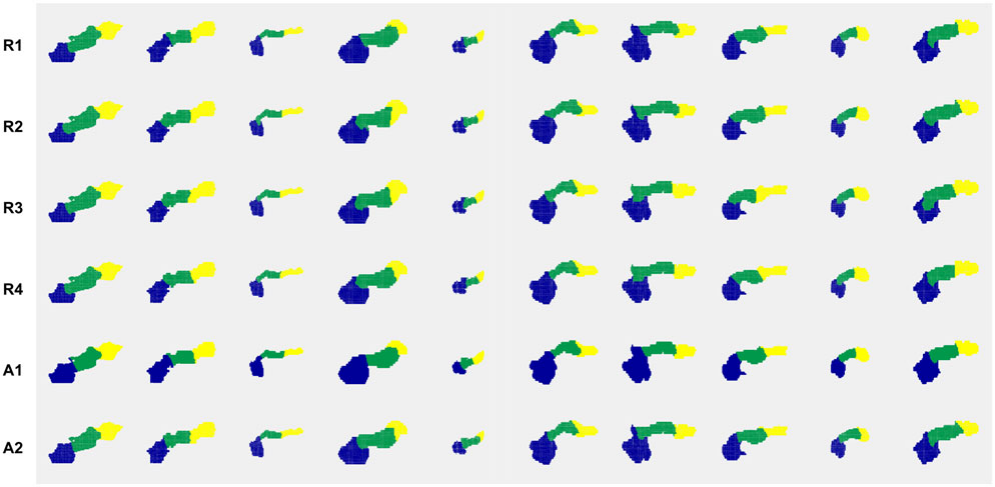
Visualization of parts segmentations from rater 1 (5 years of experience), rater 2 (25 years of experience), rater 3 (10 years of experience), rater 4 (<1 year experience), A1: Automated *k*‐means method, A2: Automated groupwise registration method, respectively (head: blue, body: green, and tail: yellow). The first 10 subjects of the validation set are shown.

Manual and automated parts segmentations for the first 10 subjects in the validation set are displayed in Fig. 4. Three‐dimensional renderings of parts segmentations from all four raters, R1–R4, including the naïve rater (R4), as well as automated parts segmentations from the *k*‐means method (A1) and the groupwise registration method (A2) are shown.

The intrarater agreement of the experienced observers R1 (R1a vs. R1b), R2 (R2a vs. R2b), R3 (R3a vs. R3b) was not significantly higher than the intrarater agreement of the naïve observer R4 (R4a vs. R4b): for R1, head: *P* = 0.3848, body: *P* = 0.2158, tail: *P* = 0.3125); for R2, head: *P* = 0.6875, body: *P* = 0.7539, tail: *P* = 0.7842; and for R3, head: *P* = 0.9033, body: *P* = 0.8838, tail: *P* = 0.2783. Similarly, the interobserver agreement between two given experienced observers (R1 vs. R2, R1 vs. R3, and R2 vs. R3 combined) was not significantly higher than the interobserver agreement between a given experienced observer vs. the naïve observer R4 (R1 vs. R4, R2 vs. R4, R3 vs. R4 combined): head: *P* = 0.9825, body: *P* = 0.9982, tail: *P* = 0.9915.

Intrarater agreement and interrater agreement were reported for each rater, separately by pancreatic head, body, and tail (Table 1). While no predefined time gap was specified between repeat annotations for intraobserver assessment, the actual time between repeat annotations varied from 2 hours later for R2 to 1 month apart for R3. Excellent intraobserver (Dice overlap, head: 0.982, body: 0.940, tail: 0.961, *N* = 30) as well as interobserver (Dice overlap, head: 0.968, body: 0.905, tail: 0.943, *N* = 150) agreement was observed in terms of segmentation performance for the combined R1, R2, and R3 metrics.

**TABLE 1:** Direct Validation Metrics, Raters 1–4 Separately and Raters 1–3 Combined

	DSC			95% HD			ΔVolume (mL)		
	Head	Body	Tail	Head	Body	Tail	Head	Body	Tail
Intrarater agreement (*n* = 10)									
R1a vs. R1b	0.988 ± 0.008	0.949 ± 0.027	0.967 ± 0.020	0.200 ± 0.632	2.096 ± 1.888	1.766 ± 1.910	−0.15 [−1.81, 1.50]	−0.90 [−2.78, 0.98]	1.06 [−1.75, 3.86]
R2a vs. R2b	0.980 ± 0.013	0.935 ± 0.030	0.950 ± 0.037	0.592 ± 1.327	3.881 ± 2.776	3.276 ± 3.068	0.05 [−2.30, 2.40]	1.00 [−4.45, 6.45]	−1.05 [−5.96, 3.86]
R3a vs. R3b	0.977 ± 0.019	0.936 ± 0.041	0.967 ± 0.028	1.036 ± 1.803	2.824 ± 2.410	1.656 ± 2.324	0.68 [−1.12, 2.49]	0.15 [−3.24, 3.54]	−0.83 [−3.57, 1.90]
R4a vs. R4b	0.982 ± 0.022	0.948 ± 0.027	0.964 ± 0.026	0.890 ± 1.636	2.636 ± 2.005	2.226 ± 2.876	0.34 [−1.39, 2.08]	−0.13 [−2.71, 2.45]	−0.22 [−2.77, 2.34]
R1, R2, R3 combined	0.982 ± 0.014	0.940 ± 0.033	0.961 ± 0.029	0.609 ± 1.342	2.934 ± 2.420	2.232 ± 2.509	0.19 [−1.83, 2.21]	0.08 [−3.95, 4.12]	−0.28 [−4.25, 3.70]
Interrater agreement (*n* = 50)									
R1 vs. R2	0.964 ± 0.021	0.909 ± 0.040	0.948 ± 0.037	2.737 ± 2.863	5.924 ± 3.365	3.220 ± 3.138	1.72 [−1.01, 4.44]	−2.61 [−7.61, 2.39]	0.89 [−3.35, 5.14]
R1 vs. R3	0.968 ± 0.023	0.907 ± 0.064	0.953 ± 0.043	2.404 ± 3.021	5.334 ± 4.626	3.223 ± 4.574	1.33 [−1.61, 4.27]	0.09 [−3.32, 3.50]	−1.42 [−4.96, 2.12]
R2 vs. R3	0.973 ± 0.020	0.898 ± 0.042	0.927 ± 0.041	1.372 ± 2.128	6.584 ± 3.994	5.633 ± 4.113	−0.39 [−3.09, 2.31]	2.70 [−1.44, 6.85]	−2.31 [−6.14, 1.52]
R1, R2, R3 combined	0.968 ± 0.022	0.905 ± 0.050	0.943 ± 0.042	2.171 ± 2.743	5.947 ± 4.033	4.026 ± 4.121	0.88 [−2.42, 4.19]	0.06 [−5.93, 6.05]	−0.95 [−5.63, 3.73]

DSC and 95% HD in mm are reported as mean ± SD. Part volumes differences are reported in mL as bias (lower LoA, upper LoA). Intraobserver agreement and interobserver agreement are reported.

DSC = Dice similarity coefficient; 95% HD = 95th percentile Hausdorff distance; LoA = limits of agreement.

“Manual vs. automated” differences in DSC, 95% HD, and volumes were reported combined across raters 1–3 for both the *k*‐means method and the groupwise registration method (Table 2). A statistically significant difference was found between “manual vs. *k*‐means method” agreement and “manual vs. groupwise registration method” agreement for the head and body segments, using DSC, but not for the tail (*P* = 0.6237).

**TABLE 2:** Direct Validation Metrics, Raters 1–3 Combined

	*k*‐Means Method vs. Manual			Groupwise Registration Method vs. Manual		
	DSC	95% HD (mm)	ΔVolume (mL)	DSC	95% HD (mm)	ΔVolume (mL)
Manual vs. auto agreement (*n* = 50)						
Head	0.942 ± 0.045	6.196 ± 5.096	−1.98 [−7.75, 3.79]	0.965 ± 0.026	2.798 ± 3.755	−0.63 [−4.46, 3.20]
Body	0.855 ± 0.120	8.899 ± 5.855	0.59 [−4.50, 5.69]	0.893 ± 0.058	6.397 ± 3.890	0.08 [−5.69, 5.85]
Tail	0.934 ± 0.054	4.870 ± 5.165	1.39 [−4.22, 7.00]	0.934 ± 0.048	4.680 ± 4.130	0.55 [−4.81, 5.91]

DSC and 95% HD in mm are reported as mean ± SD. Part volumes differences are reported in mL as bias (lower LoA, upper LoA). Manual vs. automated agreement is reported for each of the existing automated methods.

DSC = Dice similarity coefficient; 95%HD = 95th percentile Hausdorff distance; LoA = limits of agreement.

“Manual vs. *k*‐means method” agreement from Table 2 was significantly different to inter‐rater agreement from Table 1 in the head and body, using DSC, but not for the tail (*P* = 0.3965). No significant difference was found between “manual vs. groupwise registration method” agreement from Table 2 and interrater agreement from Table 1, using DSC (head: *P* = 0.4358, body: *P* = 0.0992, tail: *P* = 0.1080).

### Indirect Validation

Thirty‐eight of the validation set subjects (76%) had available multiecho gradient echo data that enabled PDFF measurement. Note that, since the pancreatic PDFF scan is single‐slice acquisition, the pancreatic head will not always be present in the image due to variable slice positioning. Similarly, when the slice position is too low, the pancreatic tail will not be visible. After processing and QC, a total of 14 subjects with visible pancreatic head, 34 with visible body, and 29 with visible tail remained for quantification.

**FIGURE 5:**
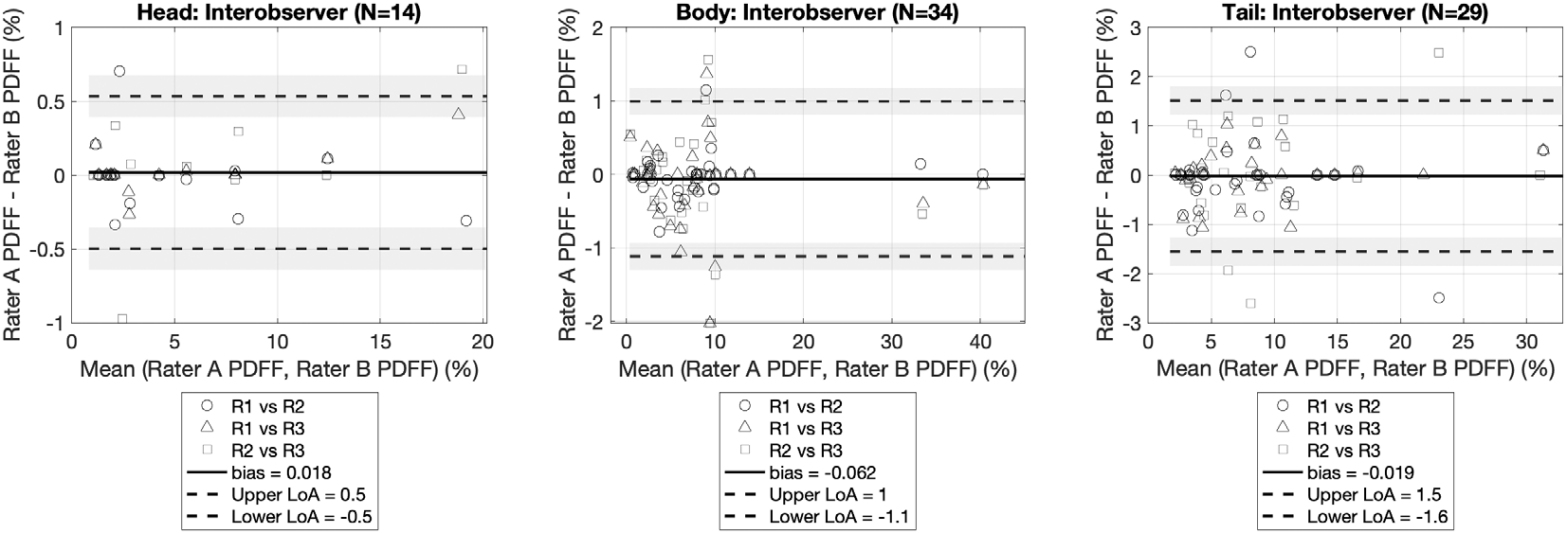
Interobserver variation of derived PDFF quantification from the manual experts' annotations. PDFF = proton density fat fraction

**FIGURE 6:**
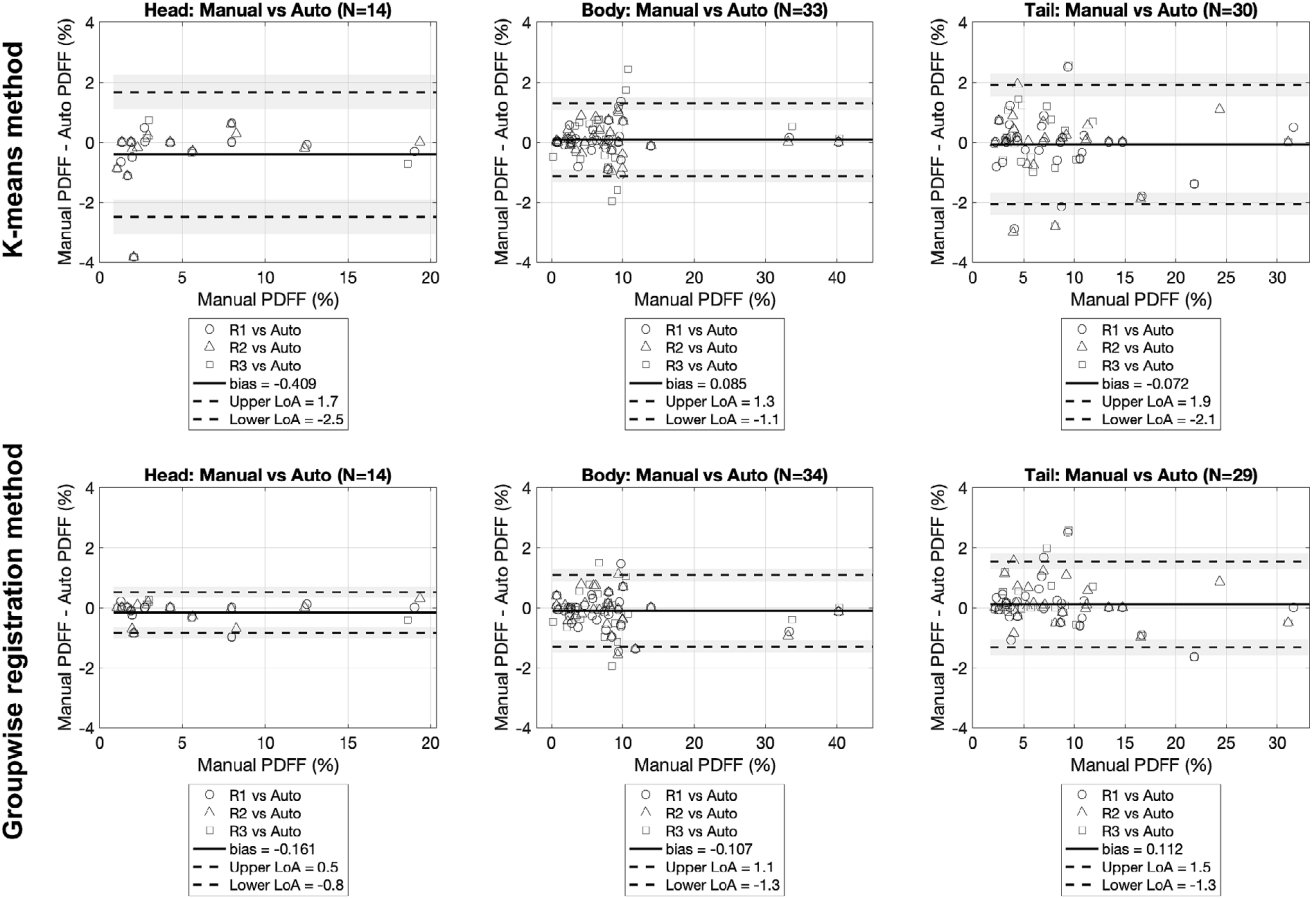
Manual experts' annotations vs. automated subsegmentations derived PDFF quantification: differences by parts. “Manual vs. auto” comparisons are presented for both the *k*‐means method and the groupwise registration method. PDFF = proton density fat fraction

Excellent interobserver agreement in PDFF quantification was observed in the Bland–Altman comparisons by pancreatic segment, as shown in Fig. 5 (head: bias = 0.018, limits of agreement [LoA] = [−0.5, 0.5]; body: bias = −0.062, LoA = [−1.1, 1.0]; tail: bias = −0.019, LoA = [−1.6, 1.5]). Both automated segmentation methods, the *k*‐means method, and the groupwise registration method showed comparable PDFF quantification agreement to the interobserver comparisons (Fig. 6). No significant differences were found between PDFF quantification from the *k*‐means method and PDFF quantification from the groupwise registration method, reported separately by parts (head: *P* = 0.5186, body: *P* = 0.1313, tail: *P* = 0.5841).

**FIGURE 7:**
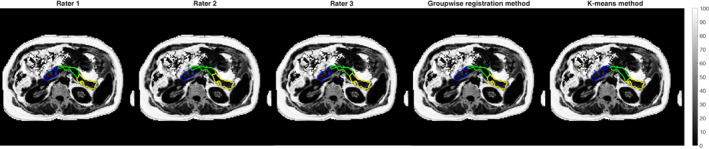
Example of regional pancreatic fat quantification for one subject in the validation set. The three‐dimensional parts segmentations for each of rater 1, rater 2, rater 3, groupwise registration method, and *k*‐means method (respectively) were separately intersected with the computed PDFF map and are shown overlaid (head: blue, body: green, tail: yellow). PDFF = proton density fat fraction

An example of a subject's PDFF map with the resliced parts segmentations from rater 1, rater 2, rater 3, the groupwise registration method, and the *k*‐means method is shown in Fig. 7.

### Pancreatic Fat Quantification by Parts in Type 2 Diabetes

**FIGURE 8:**
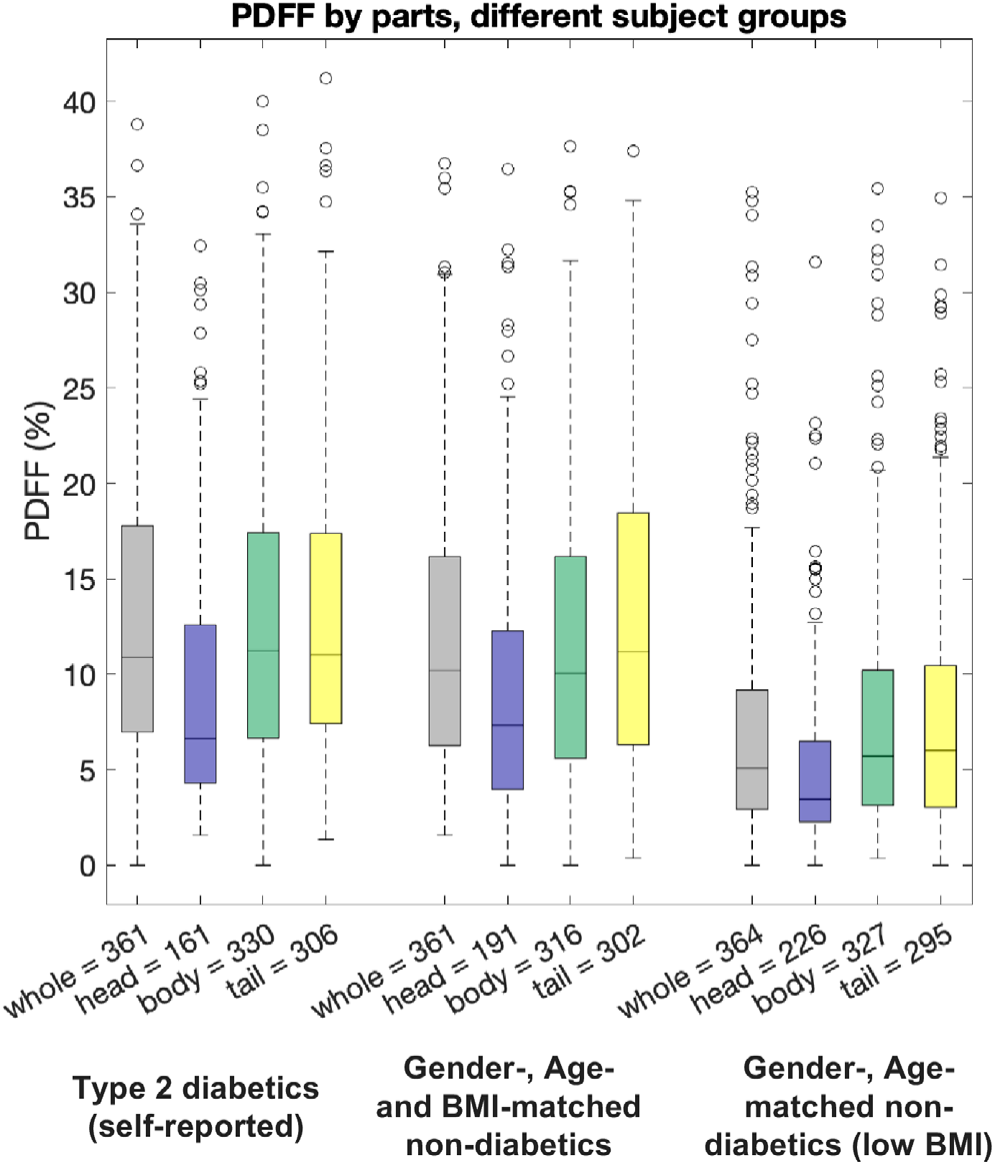
Pancreatic fat quantification by parts in groups from UK Biobank: (1) Type 2 diabetics (self‐reported), (2) nondiabetics matched by gender, age, and BMI, (3) nondiabetics matched by gender and age and with low BMI. The number of segments available is displayed next to the part. BMI = body mass index; PDFF = proton density fat fraction

Pancreatic fat quantification by pancreatic head, body, and tail for the three groups, 1) type 2 diabetics, 2) matched high BMI nondiabetics, and (3) matched low BMI nondiabetics, is shown in Fig. 8. Total pancreatic fat was also included, which was obtained after combining all the part labels into a single “whole” label. The difference in PDFF of parts between T2DM and matched low BMI nondiabetics was significant when comparing whole‐pancreas PDFF (means 12.5% and 7.2%, respectively), head PDFF (9.4% vs. 5.0%), body PDFF (12.8% vs. 7.4%), and tail PDFF (12.9% vs. 7.7%). The difference in PDFF of parts between T2DM and matched high BMI nondiabetics was significant when comparing body PDFF values (means 12.8% vs. 11.7%, respectively) but not significant when comparing whole‐pancreas PDFF (12.5% vs. 11.8%; *P* = 0.067; 95% CI = [11.8%, 13.3%] and 95% CI = [11.0%, 12.6%], respectively), head PDFF (9.4% vs. 9.1%; *P* = 0.943) or tail PDFF (12.9% vs. 12.7%; *P* = 0.623).

In terms of PDFF differences between parts within given subject groups, a similar pattern was observed for all three cohorts, namely significant differences were observed between head PDFF and body PDFF, as well as between head PDFF and tail PDFF, whereas the difference between body PDFF and tail PDFF was not significant in any of the groups: T2DM (*P* = 0.90072), matched high BMI nondiabetics (*P* = 0.071693), and matched low BMI nondiabetics (*P* = 0.65078).

## Discussion

This work presented and validated a fully automated method based on groupwise registration to subsegment the pancreas into its main anatomical parts: head, body, and tail. The method is based on a single population average or “template” image and a single annotation stage on the template, which yields a parts template that may be used for pancreas subsegmentation in new subjects. The method was validated against manual annotations from expert observers in subjects from the UK Biobank imaging substudy and was compared to previously proposed methodology based on *k*‐means clustering.^32^ Validation metrics included segmentation performance metrics as well as more clinically meaningful metrics like volume of parts and fat quantification by parts, which was obtained by intersecting the parts segmentations with PDFF maps. Then, as an initial exploration of the clinical value of parts segmentation, the method was applied to a separate UK Biobank cohort including type 2 diabetics (self‐reported) as well as gender‐, age‐, and BMI‐matched nondiabetic individuals, where the spatial distribution of pancreatic PDFF was evaluated.

Note that automated whole‐pancreas segmentation could have been used to generate both the template creation dataset and the validation dataset. However, using manual whole‐pancreas segmentations minimized introducing errors in the annotation of parts. This modular approach in which segmentation of the whole pancreas and the constituent parts are treated separately expedites validation of the subsegmentation method and allows for the introduction of improved whole‐pancreas segmentation methods when they become available. In the final experiment, which showed the potential of parts segmentation, automated segmentations were used.

Excellent intrarater and interrater agreement was observed among all raters for the proposed head, body, and tail annotation protocol. This was true not only for the three experienced raters (R1, R2, and R3) but also for the “naïve” rater (R4), suggesting that the annotation protocol is robust and repeatable and can be deployed by raters with a wide range of experience. High interobserver agreement facilitates (rather than discourages) automation, because it ensures consistent training labels for a specific task.

Most literature quantifies imaging biomarkers by head, body, tail,^20^, ^21^, ^24^ as in the work presented here, although some researchers have considered the pancreatic neck separately in the quantification.^19^ Considering the neck as an additional segment during annotation, for instance, by subdividing the head further into head and neck, could lead to increased interrater variation. In any case, considering the image resolution of the PDFF map in UK Biobank, the pancreatic neck area would be comprised of few pixels, diminishing the reliability of neck PDFF quantification. Other acquisitions and applications may be more suitable for separate neck quantification, which we will revisit in future work. Other pancreas subsegmentation systems, for instance, those incorporating embryological basis,^22^, ^23^ should also be considered in the future, for they may provide complementary regional assessment of the pancreas.

Excellent agreement was observed between the manual annotations and the automated groupwise registration method, in terms of segmentation performance and derived PDFF quantification. Significant differences were observed between manual raters and the automated *k*‐means method at partitioning pancreatic head and body, although these did not significantly impact derived PDFF quantification. The agreement between expert raters' and the automated methods' quantification suggests that the latter may be used in databases like the UK Biobank, where manual annotation is too costly or infeasible. Automation also reduces friction for a method's deployment into a clinical setting.

The *k*‐means method has the advantage of being unsupervised; however, the surrogate identification of pancreatic segments through clustering may not align well with the actual anatomical definition, compared to, for instance, using a template like in the groupwise registration method. This could explain the observation that the *k*‐means method was overestimating the head segment in the qualitative comparison. One other advantage of groupwise registration methods is that they may be used for subsequent statistical analysis of biological variation across the population. Furthermore, since the three‐dimensional parts segmentations themselves could eventually provide clinically important information, for instance, individual pancreatic segment volumes, direct segmentation performance metrics are important, for which the *k*‐means method did not provide comparable results to manual annotations. For these reasons, the groupwise registration method was used in the subsequent experiment, which characterized regional quantification of fat in type 2 diabetics.

As an initial exploration of the clinical application of our parts segmentation, we considered three matched groups: self‐reported T2DM subjects, BMI‐matched nondiabetics (mean BMI: 31.0 kg/m^2^), and age‐ and gender‐matched nondiabetics with low BMI (mean BMI: 23.0 kg/m^2^). The significantly higher whole‐pancreas PDFF in diabetics than that of nondiabetics has been reported previously.^25^ However, we have shown that PDFF in the pancreatic body is significantly different between T2DM and BMI‐matched nondiabetics, demonstrating the potential importance of parts segmentation beyond whole‐pancreas measurements, which may obscure subtle but clinically important differences. One other study showed PDFF in the pancreatic tail to be most predictive for T2DM development within 4 years.^20^ Our finding needs to be examined in more detail in future validation, eg, using dedicated T2DM cohorts with longitudinal follow‐up. The significant differences in pancreatic fat content between the pancreatic parts reported in this study emphasizes the importance of segmentation‐based approaches over ROI protocols, which should at least be “balanced” when used, meaning they should target all pancreatic segments, for instance, using multiple slices at different positions.

One method simplification could be introduced based on detecting the body–tail boundary using the pancreas segmentation centerline: the midpoint in length between the head–body boundary and the tip of the pancreatic tail would define the body–tail boundary more similar to the anatomical definition used in this work, that is, the midpoint of the total length of the body and tail, from the work of Suda et al.^22^ We may also choose to fit each predicted boundary to a plane, similar to the planes drawn in manual annotation, that is orthogonal to the pancreas centerline; in this scenario, the scalar distance between the manual boundary and predicted boundary planes may be used as the validation endpoint.

To date, we have studied regional differences in pancreatic PDFF, but note the method that is suited to report differences in other biomarkers, such as T_1_, so long as the corresponding parametric maps are available within the imaging session.

### Limitations

While our PDFF reconstruction accounted for major confounders, such as $R_{2}^{*}$ decay, multipeak fat modeling, and phase errors (although some T_1_ bias remained owing to the flip angle), the two‐dimensional nature of the PDFF scan created some limitations, namely some pancreatic segments were not visible on the two‐dimensional PDFF map for a given subject; in such cases, only the visible segments were included in further statistical analysis. Most frequently, the pancreatic head was not visible on the two‐dimensional PDFF map, which yielded an unpaired, imbalanced dataset of segmental PDFF values. This also weighted “whole” PDFF quantification toward the body and tail PDFF, relative to the head PDFF. Moreover, the fact that PDFF came from a separate breath‐hold scan may have introduced unwanted misalignment between the three‐dimensional segmentation and the PDFF map leading to PDFF quantification. The two‐point Dixon scan from the UK Biobank readily provided three‐dimensional water and fat images (with which fat fraction may be computed); however, the presence of the mentioned confounders, as well as potential fat‐water swap artifacts, discouraged their use for regional pancreatic fat quantification. The lower resolution of two‐point Dixon also complicates any postprocessing steps that are taken to avoid surrounding structures that spuriously affect fat quantification. In the future, three‐dimensional multiecho GRE acquisitions could be set up for simultaneous pancreas segmentation and confounder‐corrected three‐dimensional PDFF mapping, which would partially address these concerns.

One criticism of templates is that they might average out differences between subjects. An approach that considers multiple templates based on major components of variation may be useful, eg, clinical metadata information or imaging‐based and radiomics features.^30^ However, this increases the number of templates that need separate annotation. Evidently, in the extrema of this approach sit MAS methods and DL methods, for which individual subjects in the training set need manual annotation of parts.^34^ Our approach seemed to balance well both performance and annotation efficiency and also may generalize more robustly to various scan settings, compared to, for instance, DL methods. The template method's segmentations on the subjects it was trained with may provide good estimations of subsegmentations that could be used if labeling individual subjects is required, eg, in MAS or DL methods, speeding the annotation process. The agreement observed between expert annotations and our automatic method supports this claim.

One limitation of applying our method on type 2 diabetics is that the method was developed on UK Biobank data comprising nominally healthy volunteers aged 50–70 with no self‐reported diabetes of any type. Applying the method to the type 2 diabetes cohort might impair method performance and needs more careful evaluation. We plan to expand the method development cohort in a future version.

## Conclusion

This study demonstrated the feasibility of automated pancreas parts segmentation and downstream pancreatic imaging biomarker quantification by using groupwise registration of whole‐organ segmentations to a template and subsequent annotation of the template image. This enables segmental characterization of heterogeneous pancreatic disease.
